# Bridging the biomass data gap: A literature-based Length-Weight Relationship framework for estimating representative dry weights of freshwater invertebrates in Korean rivers

**DOI:** 10.1371/journal.pone.0352157

**Published:** 2026-06-23

**Authors:** Jaehoon Yeom, Minji Kim, Sang Don Kim

**Affiliations:** 1 Department of Civil, Environmental, and Architectural Engineering, Korea University, Seoul, Republic of Korea; 2 School of Environment and Energy Engineering, Gwangju Institute of Science and Technology, Gwangju, Republic of Korea; Swedish University of Agricultural Sciences: Sveriges lantbruksuniversitet, SWEDEN

## Abstract

Representative species weight is a critical ecological index for modeling, vulnerability assessment, and toxicity prediction, yet scientifically validated data for aquatic invertebrates remain limited. To address this gap, we present the first literature-based strategy to estimate and validate representative dry weights of freshwater invertebrate species in Korean rivers using Length-Weight Relationships (LWRs). Species length and dry weight records were compiled from domestic field guides, while dry weight records were compiled from both domestic and global literature. LWR coefficients (a and b) were then calculated at genus, family, and order levels and preprocessed under control conditions. Among the taxonomic levels tested, averaging genus-level coefficients yielded the highest concordance with field-measured dry weights (R^2^ = 0.6633, n = 240), outperforming broader taxonomic levels. Furthermore, a logarithmic correlation analysis confirmed that greater numbers of LWR sources improve predictive accuracy, particularly at the genus level. Based on this optimal strategy, representative dry weights were estimated for 563 taxa. This methodology fills a critical data gap by leveraging existing literature to generate reliable species-specific weight indices without additional field measurements. Our approach provides a quantitative foundation for biomass estimation in data-limited freshwater ecosystems and supports improved ecological modeling, conservation planning, and machine learning-based impact prediction.

## Introduction

Although systematic scientific concern regarding environmental integrity is frequently associated with the late 20th century, its modern foundations were solidified during the ‘Environmental Revolution’ of the 1960s, notably through works such as Rachel Carson’s Silent Spring (1962) [1]. Contemporary freshwater ecosystems face a multifaceted array of global change drivers, including hydrological alterations, invasive species proliferation, and intensive land-use practices. Within the specific context of South Korea, the historical legacy of rapid industrialization coupled with intensifying climatic fluctuations has established climate-driven habitat shifts and chemical perturbations as particularly critical stressors for aquatic biodiversity [2–13]. These persistent and cumulative perturbations underscore the urgent need for comprehensive research into ecosystem conservation and the refinement of environmental impact assessment (EIA) frameworks [14–16]. A fundamental challenge in advancing these frameworks lies in the scarcity of quantitative biomass data for aquatic invertebrates, which serve as essential baseline inputs for ecological models and vulnerability assessments.

Vulnerability assessments and ecological modeling serve as important tools for environmental impact assessments targeting these ecosystems [14,15,17–20]. These methods have been developed and utilized to consider complex ecological interactions, surpassing standard population-level toxicity benchmarks such as the median lethal concentration (LC_50_). By definition, LC_50_ quantifies acute mortality within a specific test population under controlled conditions, yet it lacks the capacity to inherently reflect system-scale risks or community-level responses [18,20,21]. These methods are employed when conducting impact assessments at or above the community level, serving as tools for ecological research that reflect various environmental perturbations [14,17–19]. However, such modeling requires additional ecological information including biomass of species within an ecosystem [14,22]. Therefore, the appropriate calculation of representative weight for species is one of the important tasks in ecosystem conservation research.

This study primarily focuses on generating representative weight of aquatic invertebrates in freshwater ecosystems since aquatic invertebrates are crucial biological group that plays an intermediary role in nutrient cycling and organic matter processing [23]. Among the ecosystems studied, freshwater ecosystems are crucial subjects in ecosystem conservation research because they host more than 10% of all species and one-third of vertebrate species [24]. Furthermore, freshwater ecosystems encompass a vast range of environments with unique ecological characteristics [16,24]. Consequently, constructing biomass information on freshwater invertebrates challenging due to their diversity, but it remains an essential component of ecosystem conservation research.

Studies on aquatic invertebrate biomass are generally conducted through field surveys [25,26]. Investigations of biomass in streams are carried out using DNA analysis, kick net or dip net sampling, and subsequent analysis [26,27]. However, research primarily based on field surveys often quantify each species in terms of individual counts rather than biomass [25,28]. This approach may present challenges when utilizing existing data based on individual counts. Therefore, representative species weight can help to convert individual count data to biomass data.

In the case of Korean aquatic ecosystems, quantitative research on invertebrate biomass began in the 1960s, primarily focusing on a few lakes and the four main rivers [29]. Although the Korean Ministry of Environment conducts biannual aquatic ecosystem health assessments at approximately 3,100 monitoring sites, these surveys currently prioritize individual density and species composition [25,30]. Converting these extensive datasets to biomass-based data can significantly contribute to ecological modeling [28,31,32]. However, there is a scarcity of data for calculating representative weight for many indigenous species, except for a few well-studied taxa.

Estimating species’ representative weight typically involves compiling all available individual weight data and calculating median or mean values. However, when such data are scarce, LWRs provide a practical alternative. LWRs are widely used regression models that relate an organism’s body length to its weight using species-specific coefficients [33,34]. The relationship is generally expressed as W = aL^b^ or in logarithmic form as ln(W) = ln(a)+bln(L), where W is the weight (either wet or dry), L is the body length, and a and b are species-specific constants. This method allows conversion from easily measured length data to weight estimates. However, LWR coefficients can vary due to environmental factors and body shape differences, causing potential discrepancies between estimated and actual weights [34]. To address this, researchers recommend using the geometric or arithmetic means of LWR coefficients calculated from multiple regional studies to obtain representative values [34]. Since region-specific LWR data for various freshwater invertebrate species in Korean rivers are lacking, this study compiled LWR constants from international literature to estimate the body weight of species found in Korean freshwater ecosystems. We hypothesize that taxonomic specificity at the genus level provides significantly higher predictive accuracy for invertebrate biomass than broader family- or order-level aggregations, as it better captures morphological and ecological similarity.

This study estimated the representative dry weight of freshwater invertebrates using Length-Weight Relationships (LWRs) and length data collected from field guide literature. In this study, there are several goals. 1) The preprocessing methodology for LWR coefficients is verified to extrapolate lacking LWR database. 2) The representative species weights are estimated from representative LWR coefficients and representative species length data from field guide literature based on Korean species. 3) The representative species weights are compared to the measured weight of invertebrate species recorded in previous field surveys of Korean freshwater ecosystems to check for significant differences. Despite the ecological importance of freshwater invertebrate biomass, no systematic methodology currently exists for estimating representative species weights for Korean aquatic invertebrates from existing published data. This represents a critical knowledge gap, as decades of monitoring data recorded as individual counts remain unconverted to biomass, limiting their utility for ecological modeling and conservation assessments. To address this gap, we examined whether representative dry weights can be reliably estimated using preprocessed LWR coefficients derived from global literature, without the need for additional field measurements. This entirely literature-based approach is novel in its systematic compilation, preprocessing, and validation of LWR coefficients across multiple taxonomic levels for Korean freshwater invertebrates, and is expected to make a significant contribution to future ecological data generation and biomass-based assessments.

## Methods and materials

### Study site

**Fig 1:**
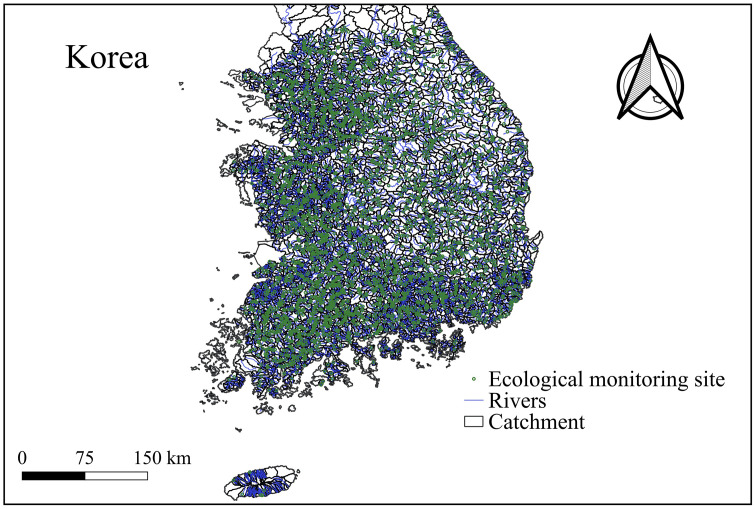
Study area and monitoring stations for ecological assessment in Korean rivers.

This study was conducted in Korean rivers, which exhibit significant ecological diversity in their freshwater ecosystems. Korea is characterized by distinct four seasons, which contribute to a high biodiversity within a relatively small geographical area. This unique feature allows for the coexistence of various species in close proximity [35]. Korea maintains approximately 3,100 ecological monitoring stations where Korean ecologists conduct biannual assessments of aquatic ecosystem health (Fig 1). Several monitoring stations for branch streams are monitored every three years, whereas the main streams are monitored annually [25]. Assessments of aquatic ecosystem health were evaluated for these monitoring stations during 2009 to present biannually. This study examined the invertebrate species monitored in Korean rivers from 2010 to 2020. The research utilized the web database of riverside monitoring points available through the Water Environment Information System (https://water.nier.go.kr/). The dataset for invertebrate species in Korean rivers was organized for all monitoring stations except for mollusk species.

### Ethical statement

No specific permits were required for this study as it was based entirely on the synthesis of data from existing literature and published field guides. Since no primary field sampling or animal collection was conducted by the authors, field site access approval was not applicable.

### Collection and conversion of individual biomass and length data of invertebrate species in Korean rivers

**Fig 2:**
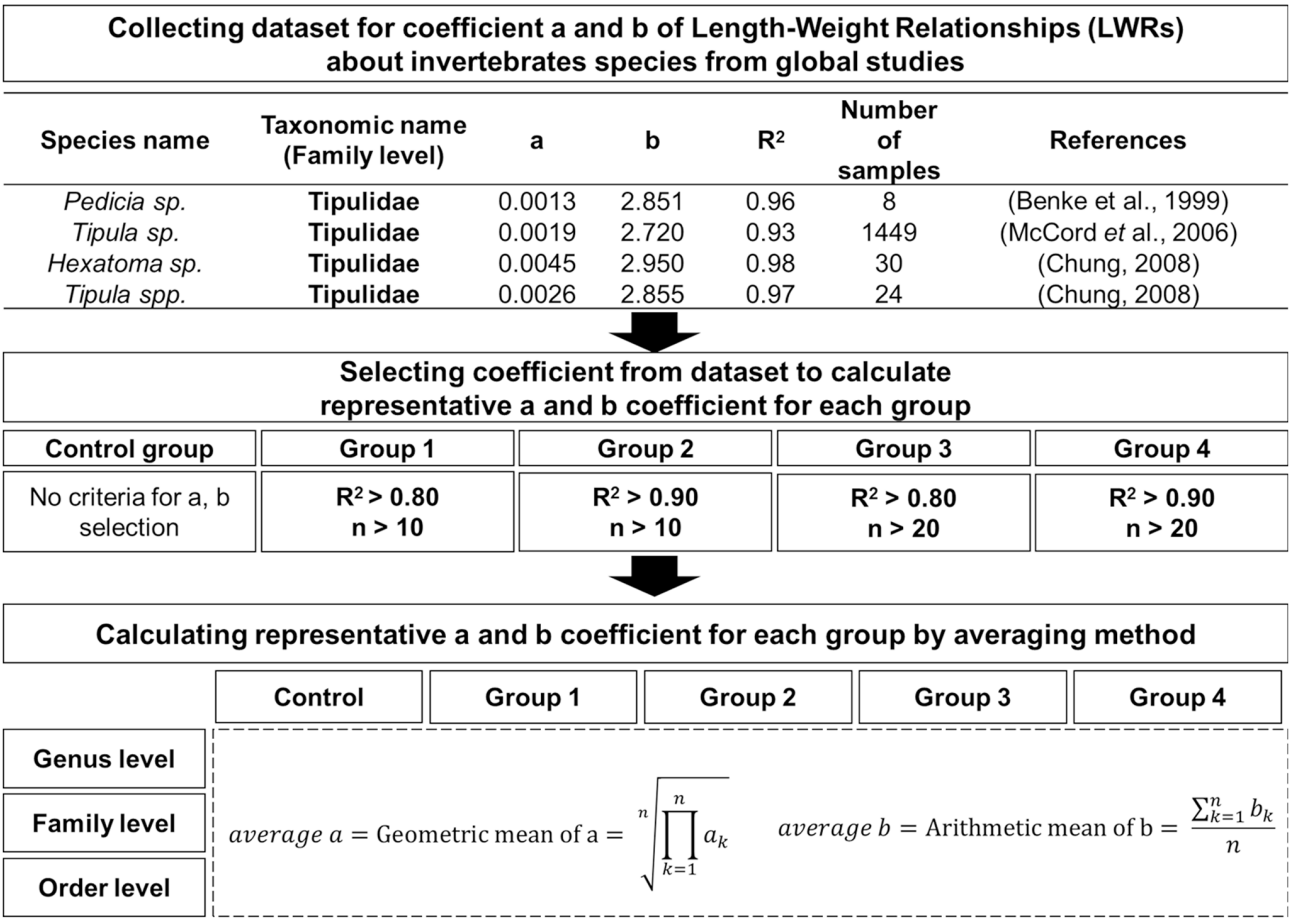
Methodology for calculating representative Length-Weight Relationships (LWRs) coefficient a and b of aquatic invertebrate species.

This study aimed to calculate the representative species weight of aquatic invertebrates inhabiting Korean rivers based on literature to contribute ecological conservation and management. To achieve this goal, the study determined the general lengths of individual species using data from various sources, including the field guide for Korean aquatic invertebrates, online databases such as National Institute of Biological Resources, and taxonomic research papers for the species included in the established database (Fig 2). For several taxonomic groups at the order level, such as Odonata and Plecoptera, where adult forms are not typically aquatic, the study recorded the average lengths based on larval stages. This approach was adopted because larvae have a more direct impact on aquatic ecosystems.

Also, the mean dry weight of each invertebrate species living in Korean rivers was evaluated by investigating the freshwater ecology in Korea. Studies from theses, domestic journals, and conferences were used to construct a database of the mean dry weights of the invertebrate species [36–41]. The individual dry weight recorded from the literature was determined by dividing the weight density by the population density because the weight data presented in the literature typically shows weight density and population density simultaneously rather than individual weight directly. Specifically, the mean wet weight of *Hydropsyche* sp. was calculated using the mean wet weight data of five size classes of larvae investigated in a previous study [36]. Because the individual weight recorded in the literature was based on wet weight, we converted it to dry weight using known wet-dry conversion ratios from the literature [42]. The conversion ratios were averaged at the order level for each taxonomic group (S1 Table). Consequently, we documented 341 dry weight data points for species across 49 genera. The abundance of data for each taxonomic group exhibits considerable variation, ranging from 1 to 55 (S1 Fig). The database was used to analyze the concordance between the measured field dry weight and the representative dry weight estimated using LWRs.

### Collecting and preprocessing of LWR coefficients for aquatic invertebrates living in Korea

The database of a and b coefficients for LWRs of invertebrate species groups living in Korea was collected from the global literature, including Korean domestic journals. To compile the global database of Length-Weight Relationship (LWR) coefficients, we aimed to cite as many academic literature sources as possible that address invertebrate length-weight relationships, regardless of their geographical origin. However, for efficiency in data collection, we prioritized scholarly papers providing datasets for multiple species (multi-species studies) rather than those focused on single individual species.

Domestic literature was searched using the Research Information Sharing Service (RISS), a comprehensive national research information sharing platform operated by the Korea Education and Research Information Service (KERIS). RISS integrates academic resources from all Korean universities and research institutions, including doctoral and master’s dissertations and journal articles published by Korean academic societies. This was supplemented by global searches in Scopus and Google Scholar using terms such as “freshwater invertebrate” and “length-weight relationship”.

While we acknowledge that intraspecific and population-level variations in body size and weight may exist across different regions and environments, no specific exclusion criteria were applied to the gathered data beyond the following predefined constant preprocessing rules. This is because the application of the geometric mean for the scaling intercept effectively generalizes these parameters and mitigates the influence of environmental or body-shape variance among different populations.

This study recorded the a and b coefficients based on dry weight-length relationships, excluding wet weight-length relationships. The coefficients a and b were incorporated into a formula that utilizes lengths in millimeters (mm) and weights in milligrams (mg). Additionally, the log-based units were converted into decimal-based units. Larval data of several species groups were selected for representative weight because adults of groups such as Odonata, Plecoptera, Diptera, and Trichoptera, which are flying insects and not aquatic organisms.

Coefficients were treated with compiling and categorizing into several groups based on coefficient conditions (R^2^ and the number of samples used for deriving coefficients (n)). The control group filtered no coefficients. The 4 experimental groups filtered coefficients by using specific criteria with R^2^ and the number of samples used for deriving coefficients (n). Group 1 included coefficients with R^2^ > 0.8 and n > 10; Group 2 included those with R^2^ > 0.9 and n > 10; Group 3 included coefficients with R^2^ > 0.8 and n > 20; and Group 4 included those with R^2^ > 0.9 and n > 20 (Fig 2).

### Calculating representative LWR coefficients for genus, family, order levels and estimating representative weight of species

The representative a and b coefficients for the LWRs were calculated by averaging coefficients belonging to the same taxonomic group at genus, family, order levels. LWR coefficients are influenced by the living habitat, morphology, and conditions around the target organisms [34]. Therefore, even for the same species, coefficients can vary depending on the study area and environmental conditions. This study generated average coefficients to generalize LWR coefficients at each taxonomic level. The representative a and b coefficients were calculated for each species using a database of coefficients collected from various regions. Coefficient a was averaged using the geometric mean as equation (1), and coefficient b was averaged using the arithmetic mean as equation (2) for the grouped coefficients [34]. The rationale for using these specific formulas lies in the statistical distribution of the LWR parameters. As established in the meta-analysis by Froese (2006), parameter a is typically log-normally distributed, necessitating the use of the geometric mean to account for this skewness and provide a central estimate. Conversely, the growth exponent b generally follows a normal distribution, making the arithmetic mean the most appropriate measure for deriving its representative value.


$$\begin{array}{c} \text{average a}=\text{Geometric mean of a}=\;\sqrt[{\text{n}}]{\prod_{\text{k}=\text{1}}^{\text{n}}{\text{a}}_{\text{k}}} \end{array}$$ (1)

$$\begin{array}{c} \text{average b}=\text{Arithmetic mean of b}=\;\frac{\sum_{k=\text{1}}^{n}b_{k}}{n} \end{array}$$ (2)

The representative dry weight of each species was calculated using the representative LWR coefficients determined for each taxonomic level and group. In the previous step, coefficients were calculated as representative values for the genus, family, and order levels with condition of the control, group 1, group 2, group 3, and group 4. Thus, each species has 15 representative LWR coefficients. Here, the variables a_ref_ and b_ref_ are defined as the representative scaling intercept and the representative growth exponent, respectively, which were derived for each specific taxonomic level (genus, family, or order) through the aforementioned averaging process. These coefficients were applied to the general total length (GTL) of the invertebrates living in Korean river systems, as recorded in field guides, to calculate the representative dry weight for each species using the following equation (3).


$$\begin{array}{c} \text{Representative species weight}={\text{a}}_{\text{ref}}{\text{GTL}}^{{\text{b}}_{\text{ref}}} \end{array}$$ (3)

The calculated representative dry weights for each species were then compared with the actual recorded weight from the field data to determine the methodology that produced the most accurate weight values. The accuracy of the estimated values was quantitatively compared using concordance R^2^ values across different groups using the following equation (4) (S2 Fig).


$$\begin{array}{c} R^{\text{2}}=\text{1}-\frac{\sum_{i=\text{1}}^{n}{\left(y_{i}-\hat{y}\right)}^{\text{2}}}{\sum_{i=\text{1}}^{n}{\left(y_{i}-\overset{―}{y}\right)}^{\text{2}}} \end{array}$$ (4)

where R^2^ is the concordance coefficient of determination used to evaluate the agreement between estimated and measured values; $y_{i}$ is the i-th field-measured dry weight; $\hat{y}$ is the corresponding estimated dry weight derived from the representative LWR coefficients; $\overset{―}{y}$ is the mean of the field-measured dry weights; and n is the total number of data points used for comparison.

The study compared 341 dry weight records of organisms from Korean freshwater ecosystem studies with the estimated representative species weight values. These estimated representative species weights were calculated using representative coefficients for length-weight relationships (LWRs) that were determined from each preprocessing strategy. During the validation of each regression model, dry weight records with Q > 6 were excluded, as suggested by Moen et al (1993). Here, $Q_{i}=\frac{\left|y_{i}\;deviation\right|}{MedAD}$ where Q_i_ is score to identify outlier values, y_i_ is each monitored dry weight value for a species, MedAD is the median of the absolute values of differences between estimated representative species weights and field measured weights [43,44]. This exclusion was necessitated by potential discrepancies in the measured weight records. These discrepancies include errors in the adult weight measurements of flying species, as reported by Kim [45].

All statistical analyses, including concordance R^2^ calculations (Eq. 4), outlier detection based on the Q-score method, and logarithmic regression analyses between the number of LWR constants and prediction accuracy with p-value test, were performed using R (version 4.4.2; R Core Team, 2024) within the RStudio integrated development environment (version 2024.10.0 + 467, Posit Software, PBC). Geometric and arithmetic means for LWR coefficients (Eqs. 1–2) were computed in Microsoft Excel, and all figures were generated using the ggplot2 package in R.

## Results

### Representative Length-Weight Relationship coefficients for Korean aquatic invertebrates across taxonomic levels

A total of 524 length-weight relationship coefficients, compiled from 23 global studies on aquatic invertebrates [37,46–66], were used to calculate representative values at multiple taxonomic levels for Korean aquatic invertebrates. When different species from the same study belonged to the same higher taxonomic level, each was treated as a separate source at that taxonomic level. Representative LWR coefficients were successfully calculated for 230 genera, 122 families, and 25 orders in the control group, with all coefficients based on body length (mm) to dry weight (mg) relationships (S2-S4 Tables).

**Fig 3:**
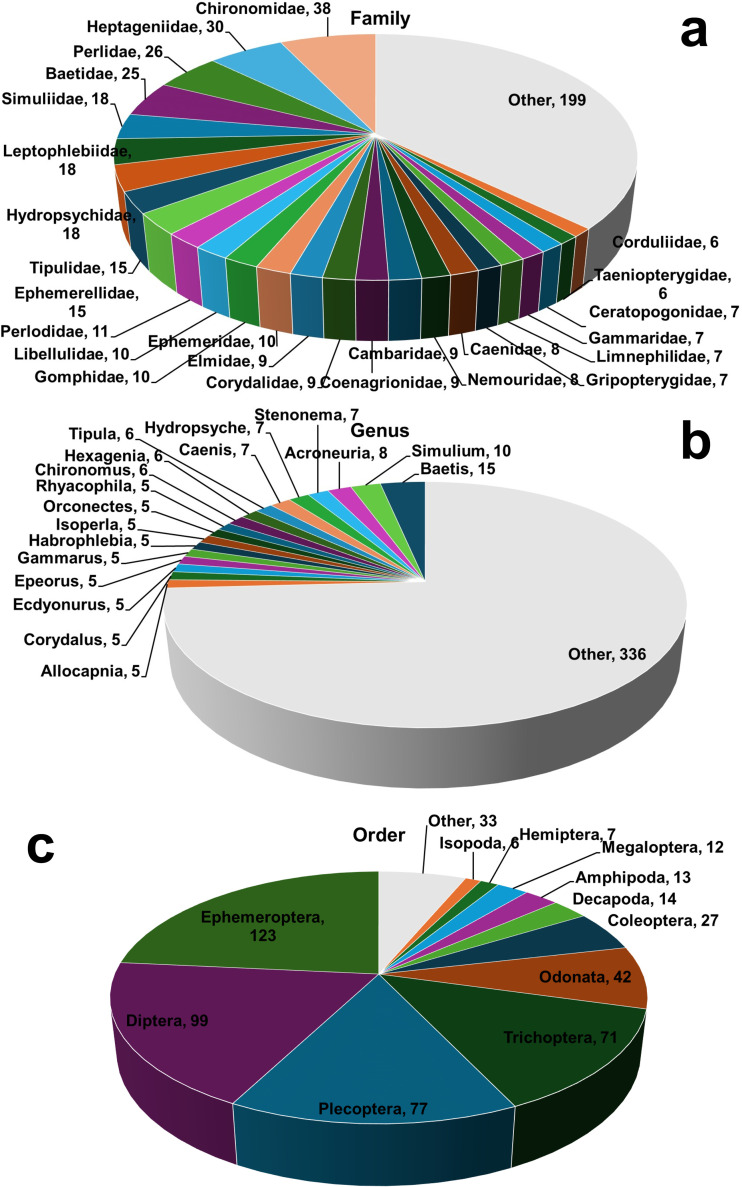
The number of Length-Weight Relationship (LWR) coefficients belonging to taxonomic groups at the (a) family level, (b) genus level, and (c) order level in database.

Genus‐level source counts ranged from single‐source genera (n = 134) to 15 coefficients for the genus Baetis, yielding a mean of 1.97 coefficients per genus (Fig 3). At the family level, 47 families had a single source coefficient, while Chironomidae was represented by 38 coefficients (mean = 4.30 per family). Order‐level coefficients varied widely, with six orders relying on a single source and Ephemeroptera represented by 123 coefficients (mean = 20.96 per order). The most extensively studied taxonomic groups were Ephemeroptera, Diptera, Plecoptera, and Trichoptera at the order level; Chironomidae, Heptageniidae, Perlidae, and Baetidae at the family level; and Baetis, Simulium, Acroneuria, and Stenonema at the genus level. These patterns highlight ecological research focus areas and data gaps in LWR study across taxonomic groups

### Representative LWR coefficients under different preprocessing criteria

The application of different preprocessing criteria significantly affected the number of taxonomic groups for which representative LWR coefficients could be calculated (S5 Table). Coverage varied substantially between taxonomic levels and preprocessing strategies. At the genus level, data availability decreased markedly with stricter criteria. Specifically, the control group achieved complete coverage (100%, n = 230), while Group 1 covered 66.70% of genera (n = 154). Groups with stricter R^2^ thresholds showed progressive reduction: Group 2 (42.61%, n = 98), Group 3 (52.17%, n = 130), and Group 4 (33.48%, n = 77). Family-level coverage ranged from 100% (control) to 40.16% (Group 4), with Group 1 achieving 70.49% representation. Order-level coverage showed the highest retention rates across all strategies, ranging from 100% (control) to 52.00% (Group 4), with Group 1 maintaining 76.00% coverage (S6 Table). Only the control group maintained 100% species coverage, while all experimental groups excluded 20% or more of target species due to insufficient qualifying data.

The validation dataset comprised 341 dry weight records representing 49 genera from Korean freshwater ecosystems. Individual data points were recorded separately for each location, time period, and source to account for environmental and individual variations in length-weight relationships. The number of data points per taxonomic group varied considerably, ranging from 1 to 55 records (S1 Fig). This variation reflects differential research attention across taxonomic groups and species abundance patterns in Korean freshwater systems. All 341 datasets included complete information for species length and wet-to-dry conversion factors where applicable. Representative dry weights were successfully calculated for the entire dataset using the hierarchical LWR coefficient application approach.

### Statistical analysis and comparison of estimation methods for calculating representative weight

**Fig 4:**
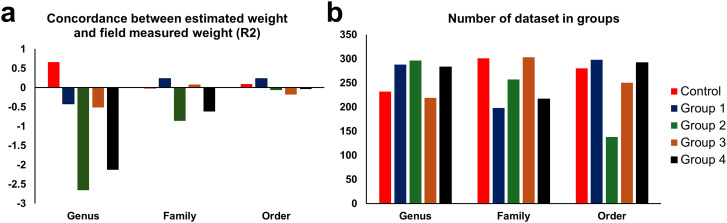
Statistical analysis of (a) R^2^ values for different taxonomic levels with varying LWR coefficients preprocessing criteria and (b) the number of datasets after excepting outliers used for evaluating R^2^ values in each group.

**Fig 5:**
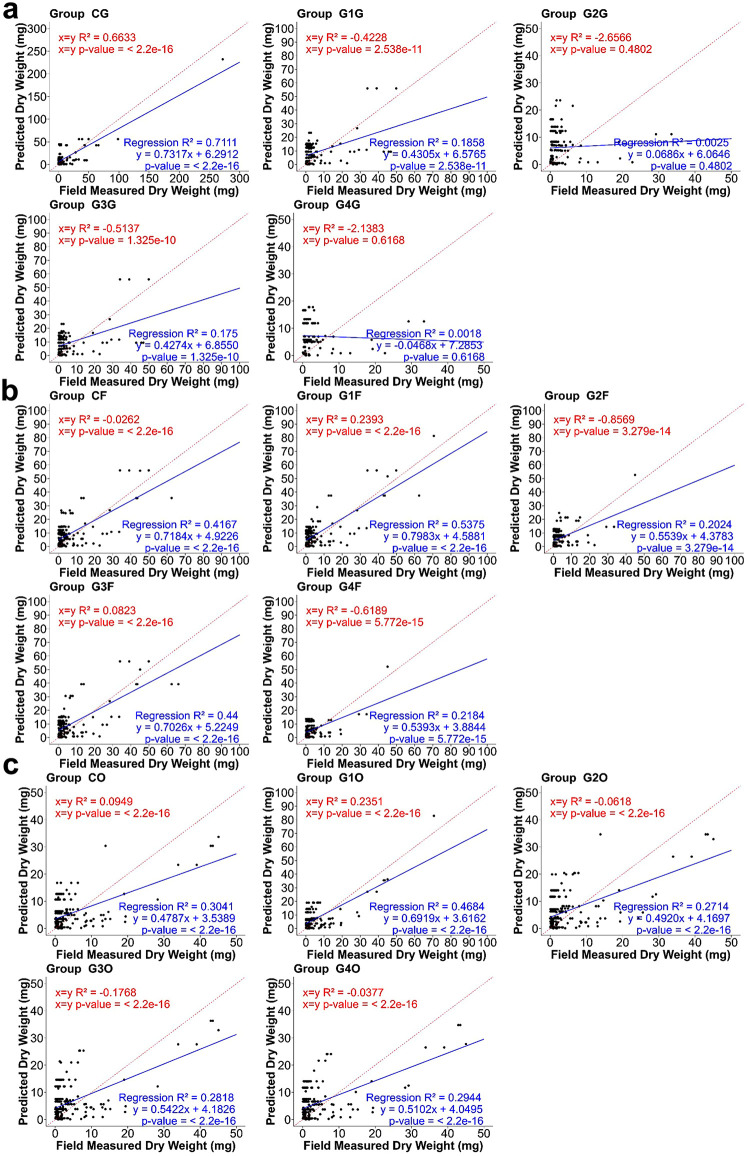
Concordance between representative species weight (mg) and field measured dry weight (mg) for preprocessing groups of LWR coefficients at (a) genus, (b) family, and (c) order level. Group C, G1, G2, G3, and G4 mean control, group 1, group 2, group 3, and group 4. G, F, and O mean genus, family, and order.

Among 15 preprocessing strategy groups tested, performance varied significantly across taxonomic levels and selection criteria (Table 1, Figs 4-5). The analysis revealed distinct patterns in concordance between estimated and field-measured weights. Among genus-level strategies, the control group achieved the highest concordance (R^2^ = 0.6633) with minimal outlier exclusion (8 points, 3.33%) (Fig 5a). Groups 1−4 showed poor model fit despite low outlier numbers. Group 1 achieved the best performance (R^2^ = 0.2393) among family-level strategies (Fig 5b). The control group showed negative concordance (R^2^ = −0.0262) with 25 outliers (7.99%). Groups 1 and 3 demonstrated positive R^2^ values, while Groups 2 and 4 showed negative performance. Group 1 was the only strategy showing positive concordance (R^2^ = 0.2351) among experimental groups at the order level (Fig 5c). The control group achieved modest positive concordance (R^2^ = 0.0949) with the highest outlier exclusion (45 points, 13.2%). All strategies achieved R^2^ values below 0.7, with genus-level control conditions demonstrating superior performance compared to all other preprocessing approaches. The number of outliers increased consistently from genus to order level across all preprocessing strategies (Table 1).

**Table 1:** R^2^ Values representing concordance between expected and observed values for 15 groups of coefficients.

	Control			Group 1			Group 2		
Coefficient group	Genus	Family	Order	Genus	Family	Order	Genus	Family	Order
**R**^**2**^ **value**	0.6633	−0.0262	0.0949	−0.4228	0.2393	0.2351	−2.6566	−0.8569	−0.0618
**Number of data point**	240	313	341	227	303	341	204	274	341
**Outlier point** **(% ratio)**	8 (3.33)	25 (7.99)	45 (13.2)	8 (3.52)	20 (6.6)	40 (11.73)	6 (2.94)	17 (6.2)	38 (11.14)
**Number of data points** **(Excepting values with Q > 6)**	232	288	296	219	283	301	198	257	303
	**Group 3**			**Group 4**					
**Coefficient group**	**Genus**	**Family**	**Order**	**Genus**	**Family**	**Order**			
**R**^**2**^ **value**	−0.5137	0.0823	−0.1768	−2.1283	−0.6189	−0.0377			
**Number of data point**	225	300	341	144	273	336			
**Outlier point** **(% ratio)**	8 (3.56)	20 (6.67)	43 (12.61)	6 (4.17)	23 (8.42)	44 (13.1)			
**Number of data point** **(Excepting values** **with Q > 6)**	217	280	298	138	250	292			

### Preprocessing requirements for representative LWR coefficients

**Fig 6:**
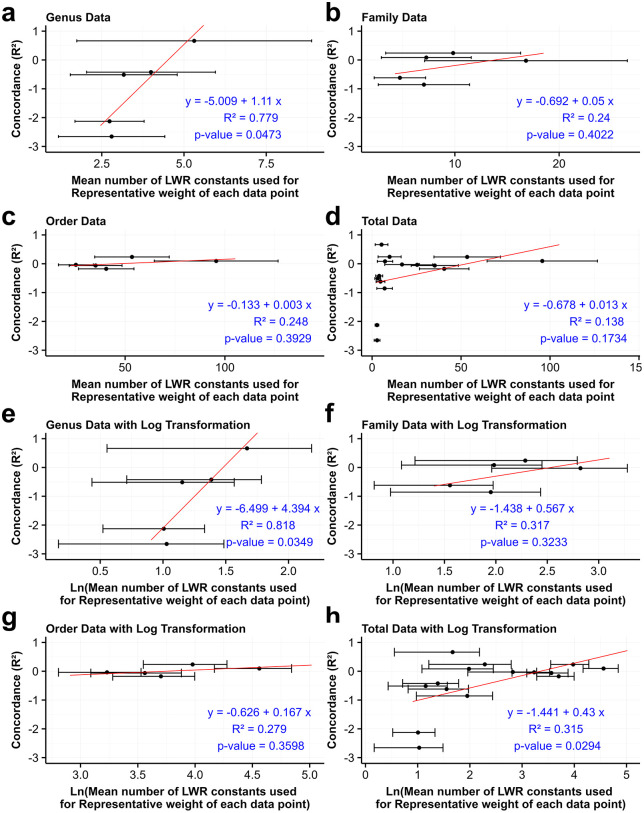
Correlation between R^2^ values and average number of data used for estimating coefficients at each taxonomic level (Genus, Family, Order); Concordance versus mean number of LWR constants used for calculating point at (a) Genus, (b) Family, (c) Order levels and (d) total dataset; Concordance versus Ln (mean number of LWR constants used for calculating point) at (e) Genus, (f) Family, (g) Order levels and (h) total dataset.

The relationship between the number of LWR constants used for representative weight calculation and prediction accuracy was analyzed across different taxonomic levels. Concordance analyses were performed comparing both linear and logarithmic relationships between the average number of LWR constants per data point and the concordance R^2^ values. The logarithmic relationship showed superior fit compared to the simple linear relationship. The best-fitting logarithmic model yielded R^2^ = 0.818 (p = 0.0349, significant explanatory power), significantly higher than the linear correlation (R^2^ = 0.779, p = 0.0473, significant explanatory power) (Fig 6a, 6e). The strength of concordance between LWR constant quantity and estimation accuracy varied significantly across taxonomic levels (Fig 6e-6h). Based on p-values (α = 0.05), significant concordances were observed only at the genus level and for the total data group. The genus level demonstrated the strongest concordance (R^2^ = 0.818) compared to the total data group (R^2^ = 0.315). Hence, the data quantity-accuracy relationship is most pronounced when taxonomic specificity is maintained at the genus level.

### Evaluation of optimal LWR pretreatment and literature-based weight estimation method

The performance of different preprocessing methods was evaluated by comparing estimated representative dry weights with field-measured values of Korean aquatic invertebrate species. Comparative analysis of preprocessing approaches revealed that genus-level control conditions achieved the highest concordance with field measurements (R^2^ = 0.6633). This performance exceeded other taxonomic level combinations and preprocessing criteria, establishing genus-level mean values as the optimal approach for representative weight estimation.

During validation, quality control procedures identified 8 data points (3.33% of total) as outliers in the genus-control group analysis (Table 1). These outliers consisted predominantly of cases where flying adult specimens were inadvertently included with aquatic samples [45]. After outlier removal, the methodology successfully explained the vast majority of field-measured weight variations (R^2^ = 0.6633). The study established representative LWR constants for 563 invertebrate species in Korean aquatic ecosystems using a hierarchical approach. The application priority followed genus-level representative constants as the primary choice, supplemented by family-level and order-level constants when genus-level data were unavailable (S1 Data). The achieved R^2^ value of 0.6633 using representative length data from field guides demonstrated comparable performance to single-species LWR studies [65,67,68], validating the effectiveness of the literature-based representative weight calculation approach for multi-species applications.

## Discussions

The compilation of LWR coefficients for aquatic invertebrates revealed substantial taxonomic biases and data quality issues inherent to existing databases. Our analysis of 23 studies showed that most taxonomic groups suffered from limited representation, with 58.26% of genera, 35.61% of families, and 24.00% of orders having only single sources [67,69–71] (S2-S4 Tables). This data scarcity particularly affects higher taxonomic levels, six order groups (Arhynchobdellida, Ixodida, Lumbriculata, Opisthorchiida, Sabellida, and Tubificida) relied on single sources (S4 Table), necessitating extrapolation of LWR constants across broader taxonomic ranges [34,66,72,73]. The limited availability of multiple sources makes it difficult to apply strict quality criteria at lower taxonomic levels, though such criteria become more feasible at higher levels where larger species pools can be evaluated. Notably, a significant finding was the ‘filtering paradox’ observed in experimental Groups 2 and 4. Despite applying stricter statistical criteria (R^2^ > 0.9), these groups showed poorer predictive accuracy (R^2^ < 0) than the unfiltered control group at the genus level. This suggests that requiring high internal correlation in source studies may lead to selection bias, where coefficients represent niche-specific populations that do not generalize well. Conversely, the aggregate mean of all available literature captures a broader range of allometric variance, making it more robust for diverse field validation data.

Our analysis revealed a fundamental trade-off between taxonomic specificity and data availability in LWR coefficient applications. The strong logarithmic correlation between the number of available LWR constants and predictive accuracy demonstrates that data quantity significantly influences estimation reliability, particularly at the genus level where ecological similarity is maximized [74–76]. However, this relationship weakens substantially at higher taxonomic levels due to increased ecological and morphological diversity within groups. While broader taxonomic aggregation provides more available data points, the resulting heterogeneity reduces the biological relevance of derived representative constants, leading to decreased predictive accuracy despite larger sample sizes [47,65].

The performance of our literature-based framework at the genus level aligns well with findings from international river systems. Our achieved concordance (R^2^ = 0.6633) for independent Korean field data is highly robust, considering these are borrowed indices. This performance sits within the range of explanatory power reported in primary studies from Japan (R^2^ = 0.51–0.83) and North America (R^2^ > 0.85). The fact that our literature-based estimates approach the precision of locally derived regressions underscores the validity of the genus-level representative strategy for regional biomass assessments [46,54]. This demonstrates that using representative genus-level constants borrowed from global literature provides a level of predictive reliability that approaches that of locally derived regressions. Furthermore, specific allometric constants show remarkable trans-continental stability; for instance, the growth exponent for Baetis in our study (b = 2.69) is highly consistent with Japanese (b = 2.72) and North American (b = 2.42–3.20) records [46,54]. This confirms that genus-level allometry is relatively stable across geographic regions.

Beyond taxonomic specificity, several ecological and methodological factors may influence the transferability of borrowed LWR constants. From an environmental perspective, lotic organisms often exhibit more streamlined body forms with lower b values as an adaptation to minimize shear stress in flowing waters [57]. Methodologically, the choice of preservative in source studies is a critical source of bias; while formalin maintains structural integrity, ethanol is known to dissolve lipids, which can reduce dry biomass estimates by up to 20% [77,78]. These variations explain why the aggregate mean approach used in our control group, which averages out such contextual noise, proved more robust than selecting high-precision constants from single, potentially niche-specific environments. Additionally, other factors such as differences in field sampling techniques (e.g., kick net vs. Surber sampler), specimen drying and measuring methods, the presence or absence of gut contents at the time of weighing, the range of body lengths measured in each study, and the sample size used for deriving coefficients can introduce systematic biases in reported LWR coefficients [77–79]. These considerations further support the rationale for aggregating coefficients from multiple sources to average out such contextual variation.

Field validation confirmed that genus-level representative constants provide the optimal balance between taxonomic specificity and data availability for biomass estimation in Korean aquatic invertebrates. The hierarchical application strategy—prioritizing genus-level constants, followed by family- and order-level constants when necessary—proved effective for practical implementation across diverse taxonomic groups. The approach using field guide-based representative lengths successfully captured field-measured weight variations, with most discrepancies attributed to developmental stage mixing rather than methodological limitations [45]. This methodology provides a robust framework for biomass estimation in data-limited contexts while maintaining biological relevance.

This study establishes a practical protocol for estimating aquatic invertebrate biomass using literature-derived LWR relationships, addressing a critical gap in ecological assessment capabilities for Korean freshwater systems. The demonstrated effectiveness of genus-level representative constants suggests that targeted research efforts should prioritize filling data gaps at this taxonomic level rather than pursuing broader taxonomic coverage. The inherent limitations of representing diverse size classes with single values highlight the need for continued field validation and expansion of species-specific databases. Future studies should focus on reducing taxonomic biases in LWR databases and developing region-specific coefficients to improve estimation accuracy for local ecological applications [66,73].

## Conclusion

This study proposed a simple and practical approach to estimate the representative dry weight of freshwater invertebrate species in Korean ecosystems using Length-Weight Relationships (LWRs). This easily applicable method reduces the dependence on time-consuming field measurements. The representative weights calculated from expert-compiled field guide data exhibited strong correlation with field-measured dry weights (R^2^ = 0.6633), with greatest accuracy achieved through genus-level constants under control conditions. This demonstrates that field guide data can function as reliable resources for ecological assessments and management.

There are, however, limitations due to insufficient LWR data for certain species, which reduces accuracy at higher taxonomic levels (family and order). Furthermore, adopting standardized methods for field sampling, laboratory processing (e.g., drying procedures, gut content removal, and body length measurement protocols), and LWR derivation would help to produce more precise and comparable coefficients across studies and regions. Further research aimed at expanding LWR databases with additional field data collected under such standardized protocols, particularly for understudied taxa, will be essential to improve method accuracy and representativeness. Additionally, future studies should apply machine learning-based approaches for more precise optimization of representative weights where data are limited. Such innovations are expected to provide more robust baseline information to support vulnerability assessments, freshwater resource management, and ecological modeling.

## Supporting information

S1 Table Wet to dry conversion ratio for orders level. (DOCX)

S2 Table Calculated representative coefficients of each taxonomic group at genus level and the number of used literatures (n) for calculating for control group. (DOCX)

S3 Table Calculated representative coefficients of each taxonomic group at family level and the number of used literatures (n) for calculating for control group. (DOCX)

S4 Table Calculated representative coefficients of taxonomic group at the order level and the number of literatures used (n) for calculating for control group. (DOCX)

S5 Table The number and ratio of calculated representative coefficients for each taxonomic group at the genus, family, and order level. (DOCX)

S6 Table Calculated representative coefficients of taxonomic group at the order level and the number of literatures used (n) for calculating for each experimental group. (DOCX)

S1 Fig The number of measured dry weight data in each taxonomic group at (a) genus, (b) family, and (c) order levels. (TIF)

S2 Fig Methodology for Evaluating Representative Biomass in Korean Invertebrates Using R^2^ Calculation. (TIF)

S1 Data Estimated representative species weight for 564 species in Korean rivers. (XLSX)
