# Supplementary material for: Bridging the biomass data gap: A literature-based Length-Weight Relationship framework for estimating representative dry weights of freshwater invertebrates in Korean rivers
Source: PLoS One. 2026 Jun 23;21(6):e0352157. doi: 10.1371/journal.pone.0352157 (PMC13289862; doi:10.1371/journal.pone.0352157)
Supplement: S1 Table — (DOCX) [file pone.0352157.s001.docx]

S1 Table. Wet to Dry conversion ratio for Orders level

| **Taxonomic name (Orders)** | **Wet to Dry ratio** |
| --- | --- |
| Coleoptera | 0.37725 |
| Amphipoda | 0.1995 |
| Cephalopoda | 0.222333 |
| Ephemeroptera | 0.140571 |
| Trichoptera | 0.185 |
| Diptera | 0.376 |
| Copepoda | 0.114 |
| Euphausiacea | 0.159 |
| Araneae | 0.35 |
| Annelida | 0.2915 |
| Plecoptera | 0.162667 |
| Oligochaeta | 0.07 |
| Gastropoda | 0.176 |
| Hemiptera | 0.365313 |
| Lepidoptera | 0.195 |
| Odonata | 0.353 |
