# Supplementary material for: Bridging the biomass data gap: A literature-based Length-Weight Relationship framework for estimating representative dry weights of freshwater invertebrates in Korean rivers
Source: PLoS One. 2026 Jun 23;21(6):e0352157. doi: 10.1371/journal.pone.0352157 (PMC13289862; doi:10.1371/journal.pone.0352157)
Supplement: S2 Table — (DOCX) [file pone.0352157.s002.docx]

S2 Table. Calculated representative coefficients of each taxonomic group at genus level and the number of used literatures (n) for calculating for control group

| **Taxonomic name**  **(Genus level)** | **n** | **a** | **b** | **Taxonomic name (Genus level)** | **n** | **a** | **b** |
| --- | --- | --- | --- | --- | --- | --- | --- |
| *Ablabesmyia* | 1 | 0.001 | 2.884 | *Ladona* | 1 | 0.0072 | 2.618 |
| *Acrocalanus* | 1 | 0.017 | 3.37 | *Lanthus* | 1 | 0.0097 | 2.895 |
| *Acroneuria* | 8 | 0.008841 | 2.869 | *Lehmannia* | 1 | 4.41E-05 | 4.69 |
| *Aedes* | 1 | 1.37E-05 | 2.82 | *Lekanesphaera* | 2 | 0.014288 | 2.465 |
| *Aegla* | 1 | 0.00627 | 3.76 | *Lepidostoma* | 2 | 0.006246 | 2.7445 |
| *Aeshna* | 2 | 0.00387 | 2.93 | *Leptophlebia* | 4 | 0.000839 | 3.4265 |
| *Agarodes* | 1 | 0.0074 | 2.741 | *Lestes* | 1 | 0.00745 | 2.97 |
| *Agnetina* | 2 | 0.02068 | 2.494 | *Leucrocuta* | 1 | 0.011 | 2.761 |
| *Allocapnia* | 5 | 0.006413 | 2.4714 | *Leuctra* | 4 | 0.003317 | 2.679 |
| *Ameletus* | 3 | 0.003933 | 2.81943 | *Libellula* | 1 | 0.0151 | 2.393 |
| *Amphinemura* | 4 | 0.009926 | 2.482493 | *Limnephilus* | 1 | 0.0015 | 3.115 |
| *Anchytarsus* | 1 | 0.0011 | 3.1 | *Limnodrilus* | 1 | 0.076083 | 0.74 |
| *Ancyronyx* | 1 | 0.0034 | 3.091 | *Limnoperla* | 1 | 0.005731 | 2.34 |
| *Antarctoperla* | 1 | 0.030669 | 1.61 | *Lipsothrix* | 1 | 6.00E-04 | 2.745 |
| *Arctopsyche* | 1 | 0.059 | 2.54 | *Lirceus* | 1 | 0.0072 | 2.785 |
| *Argia* | 2 | 0.008632 | 2.668 | *Lispoides* | 1 | 0.00033 | 3.55 |
| *Asellus* | 2 | 0.002601 | 3.3 | *Lype* | 1 | 0.0039 | 2.873 |
| *Atalophlebia* | 1 | 0.006314 | 2.564 | *Macrobrachium* | 2 | 1.12E-05 | 2.91 |
| *Atherix* | 1 | 0.0038 | 2.586 | *Macronema* | 1 | 0.003028 | 3.09 |
| *Athripsodes* | 1 | 0.014217 | 2.35 | *Macronychus* | 3 | 0.02004 | 2.534667 |
| *Aubertoperla* | 1 | 0.005135 | 2.78 | *Macrostemum* | 2 | 0.003184 | 3.0415 |
| *Austrelmis* | 1 | 0.005905 | 2.11 | *Malirekus* | 1 | 0.0119 | 2.695 |
| *Austrogomphus* | 1 | 0.002633 | 3.393 | *Meridialaris* | 1 | 4.85E-05 | 4.84 |
| *Baetis* | 15 | 0.005577 | 2.690774 | *Metamonius* | 1 | 0.001747 | 3.26 |
| *Baetisca* | 1 | 0.0116 | 2.905 | *Metrobates* | 2 | 0.016958 | 2.66 |
| *Beloneuria* | 2 | 0.008617 | 2.805 | *Micrasema* | 2 | 0.018545 | 2.5205 |
| *Blepharicera* | 1 | 0.0064 | 3.292 | *Molophilus* | 1 | 0.0016 | 2.914 |
| *Boyeria* | 2 | 0.00823 | 2.8115 | *Nemoura* | 2 | 0.003811 | 3.268515 |
| *Brachycentrus* | 2 | 0.0025 | 3.0215 | *Neoatopsyche* | 1 | 0.003883 | 2.51 |
| *Branchinecta* | 4 | 2.55E-05 | 2.85 | *Neoperla* | 2 | 0.036291 | 2.085 |
| *Bungona* | 1 | 0.008587 | 2.392 | *Neureclipsis* | 1 | 0.0023 | 2.879 |
| *Caecidotea* | 1 | 0.0036 | 3.111 | *Neurocordulia* | 1 | 0.0124 | 2.884 |
| *Caenis* | 7 | 0.00503 | 2.762571 | *Nigronia* | 4 | 0.00311 | 2.8405 |
| *Cailloma* | 1 | 0.009632 | 2.47 | *Nixe* | 1 | 0.008 | 2.919 |
| *Callibaetis* | 1 | 0.00081 | 3.547 | *Notoperla* | 1 | 0.00429 | 3.15 |
| *Calopteryx* | 1 | 0.005 | 2.742 | *Notoperlopsis* | 1 | 0.001873 | 2.94 |
| *Celithemis* | 1 | 0.0058 | 2.877 | *Nousia* | 1 | 0.007504 | 2.34 |
| *Centaurium* | 1 | 0.0918 | 2.1 | *Ochlerotatus* | 1 | 4.56E-07 | 3.97 |
| *Centroptilum* | 2 | 0.008822 | 2.7515 | *Oecetis* | 1 | 0.0034 | 3.212 |
| *Ceraclea* | 1 | 0.001295 | 4.63 | *Offadens* | 1 | 0.002373 | 2.993 |
| *Chaoborus* | 1 | 0.000453 | 2.43 | *Optioservus* | 1 | 0.0039 | 2.96 |
| *Cheumatopsyche* | 4 | 0.003611 | 2.6985 | *Orthetrum* | 1 | 0.0016 | 3.37 |
| *Chiloporter* | 1 | 0.008048 | 2.67 | *Oyamia* | 1 | 0.005508 | 3.05227 |
| *Chimarra* | 4 | 0.005194 | 2.53575 | *Pacifastacus* | 2 | 0.01452 | 3.3325 |
| *Chironomus* | 6 | 0.000479 | 2.93 | *Palpomyia* | 3 | 0.003499 | 2.308667 |
| *Cinygmula* | 1 | 0.006374 | 2.92177 | *Paragnetina* | 1 | 0.0093 | 2.797 |
| *Cladopelma* | 1 | 0.001 | 2.606 | *Paraleptophlebia* | 4 | 0.004223 | 2.89832 |
| *Clioperla* | 2 | 0.00447 | 2.8465 | *Parasericostoma* | 1 | 0.051544 | 1.45 |
| *Cloeon* | 1 | 0.001 | 3.68 | *Paratrichocladius* | 1 | 0.001712 | 3.23 |
| *Coenagrion* | 1 | 0.0248 | 2.65 | *Parvocalanus* | 1 | 0.0538 | 2.3 |
| *Cordulegaster* | 1 | 0.0067 | 2.782 | *Pedicia* | 2 | 0.000477 | 3.1805 |
| *Cordulia* | 1 | 0.0629 | 2.92 | *Peltodytes* | 2 | 0.027153 | 2.742 |
| *Corophium* | 1 | 0.0043 | 2.41 | *Penaphlebia* | 1 | 0.009072 | 2.29 |
| *Corydalus* | 5 | 0.001747 | 3.0062 | *Perlesta* | 3 | 0.003461 | 3.06 |
| *Crangonyx* | 2 | 0.003406 | 3.0045 | *Perlinella* | 1 | 0.0034 | 3.123 |
| *Culex* | 2 | 4.05E-05 | 3.09 | *Petrophila* | 1 | 0.0027 | 2.918 |
| *Cura* | 1 | 0.0101 | 2.162 | *Pictetoperla* | 2 | 0.009925 | 2.7 |
| *Dasyoma* | 1 | 0.010227 | 2.04 | *Pilaria* | 1 | 0.0014 | 2.667 |
| *Diaptomus* | 2 | 1.00E-06 | 3.5 | *Plathemis* | 1 | 0.004 | 3.068 |
| *Dicranota* | 2 | 0.00338 | 2.573365 | *Platycentropus* | 1 | 0.00919 | 2.7 |
| *Dicrotendipes* | 1 | 0.00059 | 3.142 | *Polycentropus* | 4 | 0.003711 | 2.70775 |
| *Dineutes* | 2 | 0.053212 | 2.588 | *Polypedilum* | 1 | 0.001 | 2.761 |
| *Dromogomphus* | 1 | 0.018 | 2.239 | *Pontoporeia* | 3 | 0.003755 | 3.118667 |
| *Drunella* | 4 | 0.005076 | 3.081398 | *Potamoperla* | 1 | 0.060472 | 1.38 |
| *Dugesia* | 1 | 0.0089 | 2.145 | *Procladius* | 2 | 0.000961 | 2.645 |
| *Eccoptura* | 2 | 0.002696 | 3.336 | *Progomphus* | 1 | 0.0057 | 2.831 |
| *Ecdyonurus* | 5 | 0.009242 | 2.88947 | *Promoresia* | 1 | 0.0025 | 3.521 |
| *Echinogammarus* | 1 | 0.0031 | 2.66 | *Prosimulium* | 3 | 0.001258 | 3.143333 |
| *Ecnomus* | 1 | 0.003472 | 2.433 | *Prostoia* | 2 | 0.015468 | 2.2115 |
| *Ectopria* | 1 | 0.0164 | 2.929 | *Psephenus* | 1 | 0.0077 | 2.883 |
| *Edwardsina* | 1 | 0.006724 | 2.85 | *Pseudocloeon* | 1 | 0.0023 | 3.264 |
| *Empididae* | 2 | 0.005138 | 2.5455 | *Pseudonereis* | 2 | 0.002 | 2.558 |
| *Epeorus* | 5 | 0.009391 | 2.83105 | *Psilotreta* | 3 | 0.009726 | 2.91726 |
| *Ephemera* | 4 | 0.002398 | 2.890983 | *Psychomyiidae* | 1 | 0.0234 | 1.63 |
| *Ephemerella* | 4 | 0.009823 | 2.59425 | *Pteronarcys* | 1 | 0.0064 | 2.845 |
| *Ephoron* | 2 | 0.002 | 3.05 | *Ptilostomis* | 1 | 0.0054 | 2.811 |
| *Epitheca* | 2 | 0.008161 | 2.7385 | *Pycnopsyche* | 3 | 0.005506 | 2.807333 |
| *Erythemis* | 1 | 0.0061 | 3.089 | *Pyrrhosoma* | 2 | 0.005691 | 2.865 |
| *Erythromma* | 1 | 0.107 | 2.08 | *Rhagovelia* | 2 | 0.008333 | 2.7785 |
| *Eubranchipus* | 2 | 0.000531 | 2.46 | *Rheochorema* | 1 | 0.009163 | 2.37 |
| *Euglossa* | 1 | 0.0148 | 2.21 | *Rhithrogena* | 3 | 0.005116 | 3.064667 |
| *Eurylophella* | 3 | 0.005906 | 2.515 | *Rhyacophila* | 5 | 0.007015 | 2.812766 |
| *Ficopomatus* | 1 | 0.0101 | 1.61 | *Schistocerca* | 1 | 0.005 | 1.98 |
| *Galerucella* | 1 | 0.0392 | 3.111 | *Scopula* | 1 | 0.006027 | 2.85188 |
| *Gammarus* | 5 | 0.00417 | 2.7642 | *Senzilloides* | 1 | 0.009255 | 2.7 |
| *Gerris* | 2 | 0.015048 | 2.598 | *Serratella* | 2 | 0.009521 | 2.7435 |
| *Girardia* | 1 | 0.018248 | 1.91 | *Sialis* | 4 | 0.003571 | 2.81625 |
| *Glossosoma* | 4 | 0.012815 | 2.745 | *Sigara* | 2 | 0.010883 | 2.717 |
| *Goera* | 1 | 0.025 | 2.575 | *Simulium* | 10 | 0.002253 | 2.7637 |
| *Gomphus* | 3 | 0.004893 | 3.030333 | *Siphlonisca* | 1 | 2.00E-04 | 3.61 |
| *Habrophlebia* | 5 | 0.002758 | 2.8012 | *Siphlonurus* | 2 | 0.000179 | 3.995 |
| *Haemopis* | 1 | 0.012 | 2.809 | *Smicridea* | 1 | 0.008544 | 2.79 |
| *Hediste* | 1 | 0.0023 | 2.2 | *Somatochlora* | 1 | 0.0882 | 2.85 |
| *Helianthus* | 1 | 0.0041 | 2.97 | *Stavsolus* | 1 | 0.008413 | 2.73435 |
| *Hemigomphus* | 1 | 0.010825 | 2.861 | *Stegopterna* | 3 | 0.000593 | 3.626667 |
| *Heptagenia* | 3 | 0.014158 | 2.462333 | *Stenacron* | 3 | 0.006365 | 2.799667 |
| *Heterocloeon* | 1 | 0.0052 | 2.721 | *Stenelmis* | 2 | 0.020696 | 2.55 |
| *Hexagenia* | 6 | 0.002245 | 2.9465 | *Stenonema* | 7 | 0.007805 | 2.789 |
| *Hexatoma* | 3 | 0.002025 | 2.945463 | *Stenopsyche* | 1 | 0.056 | 2.29 |
| *Hyalella* | 1 | 0.023189 | 2.31 | *Streptocephalus* | 1 | 0.2762 | 1.39 |
| *Hydatophylax* | 1 | 0.005261 | 2.91395 | *Strophopteryx* | 2 | 0.006391 | 2.6785 |
| *Hydrobasileus* | 1 | 0.095819 | 1.888 | *Sweltsa* | 2 | 0.004228 | 2.840675 |
| *Hydroporus* | 2 | 0.061931 | 2.501 | *Sympetrum* | 1 | 0.0932 | 2.73 |
| *Hydropsyche* | 7 | 0.005302 | 2.885429 | *Tabanus* | 1 | 0.005 | 2.591 |
| *Hydroptila* | 1 | 0.01224 | 2.57 | *Taeniopteryx* | 4 | 0.006991 | 2.66525 |
| *Ironoquia* | 1 | 0.0041 | 2.933 | *Tallaperla* | 3 | 0.015682 | 2.697667 |
| *Irpacaenis* | 1 | 0.005953 | 2.458 | *Tanytarsus* | 1 | 0.0012 | 2.294 |
| *Ischnura* | 1 | 0.0015 | 2.904 | *Teloganopsis* | 1 | 0.021 | 2.315 |
| *Isogenus* | 2 | 0.008032 | 2.708 | *Tenagomysis* | 2 | 0.003922 | 2.575 |
| *Isonychia* | 4 | 0.003097 | 3.04375 | *Tinodes* | 1 | 0.004885 | 2.32 |
| *Isoperla* | 5 | 0.010544 | 2.5384 | *Tipula* | 6 | 0.003301 | 2.608458 |
| *Kamimuria* | 1 | 0.006575 | 3.02688 | *Tribelos* | 1 | 0.000281 | 4.16 |
| *Kirkaldyia* | 1 | 0.023 | 2.988 | *Tricorythodes* | 3 | 0.006263 | 3.202333 |
| *Klapopteryx* | 1 | 0.005789 | 2.53 | *Tropisternus* | 1 | 0.002428 | 2.2 |
| *Laccotrephes* | 1 | 0.02 | 2.981 | *Urothemis* | 1 | 0.012314 | 2.728 |
