# Supplementary material for: Bridging the biomass data gap: A literature-based Length-Weight Relationship framework for estimating representative dry weights of freshwater invertebrates in Korean rivers
Source: PLoS One. 2026 Jun 23;21(6):e0352157. doi: 10.1371/journal.pone.0352157 (PMC13289862; doi:10.1371/journal.pone.0352157)
Supplement: S3 Table — (DOCX) [file pone.0352157.s003.docx]

S3 Table. Calculated representative coefficients of each taxonomic group at family level and the number of used literatures (n) for calculating for control group

| **Taxonomic name (Family level)** | **n** | **a** | **b** | **Taxonomic name (Family level)** | **n** | **a** | **b** |
| --- | --- | --- | --- | --- | --- | --- | --- |
| Acrididae | 2 | 0.020616 | 2.127 | Heptageniidae | 30 | 0.008154 | 2.825479 |
| Aeglidae | 1 | 0.00627 | 3.76 | Heterophyidae | 1 | 0.001747 | 3.26 |
| Aeshnidae | 4 | 0.005644 | 2.87075 | Hidrophilidae | 1 | 0.002428 | 2.2 |
| Ameletidae | 3 | 0.003933 | 2.81943 | Hyalellidae | 1 | 0.023189 | 2.31 |
| Ameletopsidae | 1 | 0.008048 | 2.67 | Hydrobiosidae | 3 | 0.006998 | 2.45 |
| Aphodiidae | 1 | 0.0066 | 3.18 | Hydrophilidae | 1 | 0.0275 | 2.39 |
| Apidae | 1 | 0.006 | 3.407 | Hydropsychidae | 18 | 0.005839 | 2.82 |
| Asellidae | 4 | 0.003639 | 3.124 | Hydroptilidae | 1 | 0.01224 | 2.57 |
| Asilidae | 1 | 0.38 | 1.5 | Isonychiidae | 4 | 0.003097 | 3.04375 |
| Astacidae | 2 | 0.01452 | 3.3325 | Ixodidae | 1 | 0.017 | 3.37 |
| Athericidae | 4 | 0.014533 | 2.208 | Lepidostomatidae | 2 | 0.006246 | 2.7445 |
| Austroperlidae | 1 | 0.005789 | 2.53 | Leptoceridae | 3 | 0.00397 | 3.397333 |
| Baetidae | 25 | 0.003883 | 2.878224 | Leptophlebiidae | 18 | 0.00225 | 2.998793 |
| Baetiscidae | 1 | 0.0116 | 2.905 | Lestidae | 1 | 0.00745 | 2.97 |
| Belostomatidae | 1 | 0.023 | 2.988 | Leuctridae | 4 | 0.003317 | 2.679 |
| Blephariceridae | 2 | 0.00656 | 3.071 | Libellulidae | 10 | 0.011988 | 2.7249 |
| Bombyliidae | 1 | 0.007 | 3.337 | Limnephilidae | 7 | 0.004686 | 2.869136 |
| Bostrichidae | 1 | 0.039 | 2.764 | Lumbriculidae | 1 | 0.000104 | 3.25 |
| Brachycentridae | 4 | 0.006809 | 2.771 | Muscidae | 1 | 0.00033 | 3.55 |
| Branchinectidae | 4 | 2.55E-05 | 2.85 | Mysidae | 2 | 0.003922 | 2.575 |
| Caenidae | 8 | 0.005137 | 2.7245 | Nemouridae | 8 | 0.00873 | 2.61125 |
| Calopterygidae | 1 | 0.005 | 2.742 | Nepidae | 1 | 0.02 | 2.981 |
| Capniidae | 5 | 0.006413 | 2.4714 | Nereididae | 4 | 0.005274 | 2.1065 |
| Carabidae | 1 | 0.072 | 2.401 | Odontoceridae | 3 | 0.009726 | 2.91726 |
| Ceratopogonidae | 7 | 0.001507 | 2.205714 | Palaemonidae | 2 | 1.12E-05 | 2.91 |
| Chaoboridae | 1 | 0.000453 | 2.43 | Pediciidae | 1 | 0.004232 | 2.50973 |
| Chirocephalidae | 2 | 0.000531 | 2.46 | Peltoperlidae | 3 | 0.015682 | 2.697667 |
| Chironomidae | 38 | 0.001367905 | 2.649615 | Perlidae | 26 | 0.008105 | 2.838083 |
| Chloroperlidae | 3 | 0.004471 | 2.80445 | Perlodidae | 11 | 0.008504 | 2.657305 |
| Chrysomelidae | 1 | 0.0392 | 3.111 | Philopotamidae | 5 | 0.004935 | 2.577236 |
| Cicadellidae | 1 | 0.079 | 2.229 | Phryganeidae | 1 | 0.0054 | 2.811 |
| Cicadidae | 1 | 0.004 | 3.373 | Planariidae | 3 | 0.007955 | 2.168333 |
| Coccinellidae | 1 | 0.343 | 1.5 | Polycentropodidae | 5 | 0.003372 | 2.742 |
| Coenagrionidae | 9 | 0.009861 | 2.608 | Polymitarcyidae | 2 | 0.002 | 3.05 |
| Cordulegastridae | 1 | 0.0067 | 2.782 | Pontoporeiidae | 3 | 0.003755 | 3.118667 |
| Corduliidae | 6 | 0.019082 | 2.8625 | Psephenidae | 2 | 0.011237 | 2.906 |
| Corixidae | 2 | 0.010883 | 2.717 | Psychomyiidae | 3 | 0.007639 | 2.274333 |
| Corophiidae | 1 | 0.0043 | 2.41 | Pteronarcyidae | 3 | 0.055852 | 2.215 |
| Corydalidae | 9 | 0.002257 | 2.932556 | Ptilodactylidae | 1 | 0.0011 | 3.1 |
| Crangonyctidae | 2 | 0.003406 | 3.0045 | Pyralidae | 2 | 0.005692 | 2.8065 |
| Cucilidae | 1 | 0.032 | 2.038 | Rhyacophilidae | 5 | 0.007015 | 2.812766 |
| Culicidae | 3 | 2.05E-06 | 3.393333 | Sciaridae | 1 | 0.004 | 2.091 |
| Diaptomidae | 2 | 1.00E-06 | 3.5 | Sericostomatidae | 2 | 0.01953 | 2.0955 |
| Dugesiidae | 1 | 0.018248 | 1.91 | Serpulidae | 1 | 0.0101 | 1.61 |
| Dytiscidae | 2 | 0.061931 | 2.501 | Sialidae | 4 | 0.003571 | 2.81625 |
| Ecnomidae | 2 | 0.001559 | 2.5965 | Simuliidae | 18 | 0.001823 | 2.960636 |
| Elmidae | 9 | 0.009572 | 2.709556 | Siphlonuridae | 3 | 0.000185 | 3.866667 |
| Empididae | 2 | 0.005138 | 2.5455 | Sphaeromatidae | 2 | 0.014288 | 2.465 |
| Ephemerellidae | 15 | 0.00801 | 2.683973 | Sphecidae | 1 | 0.166 | 1.797 |
| Ephemeridae | 10 | 0.002305 | 2.924293 | Staphylinidae | 2 | 0.000806 | 3.798 |
| Formicidae | 1 | 0.027 | 2.666 | Stenopsychidae | 1 | 0.056 | 2.29 |
| Gammaridae | 7 | 0.004054 | 2.525857 | Stratiomyidae | 1 | 0.0032 | 2.61 |
| Geometridae | 1 | 0.006027 | 2.85188 | Streptocephalidae | 1 | 0.2762 | 1.39 |
| Gerridae | 3 | 0.015677 | 2.618667 | Tabanidae | 2 | 0.004899 | 2.6755 |
| Glossosomatidae | 5 | 0.014278 | 2.7008 | Taeniopterygidae | 6 | 0.006785 | 2.669667 |
| Goeridae | 1 | 0.025 | 2.575 | Tetrigidae | 1 | 0.358 | 1.5 |
| Gomphidae | 10 | 0.005266 | 2.9508 | Tipulidae | 15 | 0.001833 | 2.787409 |
| Gripopterygidae | 7 | 0.008792 | 2.414286 | Tricorythidae | 3 | 0.006263 | 3.202333 |
| Gyrinidae | 2 | 0.053212 | 2.588 | Tubificidae | 1 | 0.076083 | 0.74 |
| Haemopidae | 1 | 0.012 | 2.809 | Veliidae | 3 | 0.010548 | 2.739 |
| Haliplidae | 2 | 0.027153 | 2.742 | Vespidae | 1 | 0.001 | 3.723 |
