# Supplementary material for: Bridging the biomass data gap: A literature-based Length-Weight Relationship framework for estimating representative dry weights of freshwater invertebrates in Korean rivers
Source: PLoS One. 2026 Jun 23;21(6):e0352157. doi: 10.1371/journal.pone.0352157 (PMC13289862; doi:10.1371/journal.pone.0352157)
Supplement: S4 Table — (DOCX) [file pone.0352157.s004.docx]

S4 Table. Calculated representative coefficients of taxonomic group at the order level and the number of literatures used (n) for calculating for control group

| **Taxonomic name (Order level)** | **n** | **a** | **b** | **Taxonomic name**  **(Order level)** | **n** | **a** | **b** |
| --- | --- | --- | --- | --- | --- | --- | --- |
| Amphipoda | 14 | 0.004424 | 2.697571 | Lumbriculata | 1 | 0.000104 | 3.25 |
| Anostraca | 7 | 0.000229 | 2.53 | Megaloptera | 13 | 0.0026 | 2.896769 |
| Arhynchobdellida | 1 | 0.012 | 2.809 | Mysida | 2 | 0.003922 | 2.575 |
| Calanoida | 2 | 1.00E-06 | 3.5 | Odonata | 42 | 0.008979 | 2.794786 |
| Coleoptera | 27 | 0.013957 | 2.744519 | Opisthorchiida | 1 | 0.001747 | 3.26 |
| Decapoda | 5 | 0.000699 | 3.249 | Orthoptera | 3 | 0.053386 | 1.918 |
| Diptera | 99 | 0.001665 | 2.695855 | Phyllodocida | 4 | 0.005274 | 2.1065 |
| Ephemeroptera | 123 | 0.00429 | 2.890342 | Plecoptera | 77 | 0.008268 | 2.669907 |
| Hemiptera | 12 | 0.014375 | 2.7565 | Sabellida | 1 | 0.0101 | 1.61 |
| Hymenoptera | 4 | 0.012806 | 2.89825 | Trichoptera | 71 | 0.006434 | 2.73701 |
| Isopoda | 6 | 0.00574 | 2.904333 | Tricladida | 4 | 0.00979 | 2.10375 |
| Ixodida | 1 | 0.017 | 3.37 | Tubificida | 1 | 0.000104 | 3.25 |
| Lepidoptera | 3 | 0.005802 | 2.821627 |  |  |  |  |
