# Supplementary material for: Bridging the biomass data gap: A literature-based Length-Weight Relationship framework for estimating representative dry weights of freshwater invertebrates in Korean rivers
Source: PLoS One. 2026 Jun 23;21(6):e0352157. doi: 10.1371/journal.pone.0352157 (PMC13289862; doi:10.1371/journal.pone.0352157)
Supplement: S5 Table — (DOCX) [file pone.0352157.s005.docx]

**S5 Table.** The number and ratio of calculated representative coefficients for each taxonomic group at the genus, family, and order level

| **Group name** | **Control (%)** | **Group 1 (%)** | **Group 2 (%)** | **Group 3 (%)** | **Group 4 (%)** |
| --- | --- | --- | --- | --- | --- |
| Genus group | 230  (100) | 154  (66.70) | 98  (42.61) | 130  (52.17) | 77  (33.48) |
| Family group | 122  (100) | 86  (70.49) | 63  (51.64) | 70  (57.38) | 49  (40.16) |
| Order  group | 25  (100) | 19  (76.00) | 16  (64.00) | 18  (72.00) | 13  (52.00) |
