# Supplementary material for: Bridging the biomass data gap: A literature-based Length-Weight Relationship framework for estimating representative dry weights of freshwater invertebrates in Korean rivers
Source: PLoS One. 2026 Jun 23;21(6):e0352157. doi: 10.1371/journal.pone.0352157 (PMC13289862; doi:10.1371/journal.pone.0352157)
Supplement: S6 Table — (DOCX) [file pone.0352157.s006.docx]

S6 Table. Calculated representative coefficients of taxonomic group at the order level and the number of literatures used (n) for calculating for each experimental group

| **Group name** | **Group 1** | | | **Group 2** | | | **Group 3** | | | **Group 4** | | |
| --- | --- | --- | --- | --- | --- | --- | --- | --- | --- | --- | --- | --- |
| **Taxonomic name (Order)** | **n** | **a** | **b** | **n** | **a** | **b** | **n** | **a** | **b** | **n** | **a** | **b** |
| Amphipoda | 11 | 0.005 | 2.766 | 6 | 0.005 | 2.841 | 0.005 | 2.755 | 0.005 | 5 | 0.006 | 2.835 |
| Anostraca | 1 | 0.276 | 1.390 | 1 | 0.276 | 1.390 | 0.276 | 1.390 | 0.276 | 1 | 0.276 | 1.390 |
| Arhynchobdellida | 1 | 0.012 | 2.809 | - | - | - | 0.012 | 2.809 | 0.012 | - | - | - |
| Calanoida | - | - | - | - | - | - | - | - | - | - | - | - |
| Coleoptera | 14 | 0.023 | 2.692 | 8 | 0.032 | 2.595 | 0.018 | 2.673 | 0.018 | 5 | 0.018 | 2.677 |
| Decapoda | - | - | - | - | - | - | - | - | - | - | - | - |
| Diptera | 54 | 0.001 | 2.805 | 34 | 0.002 | 2.893 | 0.001 | 2.873 | 0.001 | 24 | 0.001 | 3.009 |
| Ephemeroptera | 65 | 0.004 | 2.986 | 39 | 0.003 | 3.056 | 0.003 | 3.041 | 0.003 | 30 | 0.003 | 3.141 |
| Hemiptera | 7 | 0.015 | 2.689 | 2 | 0.037 | 2.445 | 0.015 | 2.772 | 0.015 | 1 | 0.017 | 2.660 |
| Hymenoptera | 1 | 0.001 | 3.723 | 1 | 0.001 | 3.723 | - | - | - | - | - | - |
| Isopoda | 4 | 0.006 | 2.802 | 2 | 0.005 | 2.818 | 0.006 | 2.802 | 0.006 | 2 | 0.005 | 2.818 |
| Ixodida | - | - | - | - | - | - | - | - | - | - | - | - |
| Lumbriculata | - | - | - | - | - | - | - | - | - | - | - | - |
| Megaloptera | 11 | 0.002 | 2.930 | 7 | 0.002 | 2.984 | 0.002 | 2.933 | 0.002 | 7 | 0.002 | 2.984 |
| Mysida | 2 | 0.004 | 2.575 | 1 | 0.003 | 2.760 | 0.004 | 2.575 | 0.004 | 1 | 0.003 | 2.760 |
| Odonata | 24 | 0.006 | 2.817 | 14 | 0.005 | 2.877 | 0.008 | 2.813 | 0.008 | 7 | 0.009 | 2.761 |
| Opisthorchiida | 1 | 0.002 | 3.260 | - | - | - | 0.002 | 3.260 | 0.002 | - | - | - |
| Orthoptera | 2 | 0.174 | 1.887 | 1 | 0.085 | 2.274 | 0.358 | 1.500 | 0.358 | - | - | - |
| Phyllodocida | 3 | 0.002 | 2.439 | 1 | 0.002 | 2.572 | 0.002 | 2.439 | 0.002 | 1 | 0.002 | 2.572 |
| Plecoptera | 50 | 0.009 | 2.683 | 27 | 0.012 | 2.540 | 0.010 | 2.608 | 0.010 | 20 | 0.015 | 2.425 |
| Sabellida | - | - | - | - | - | - | - | - | - | - | - | - |
| Trichoptera | 34 | 0.005 | 2.844 | 20 | 0.005 | 2.833 | 0.005 | 2.850 | 0.005 | 13 | 0.005 | 2.839 |
| Tricladida | 2 | 0.012744 | 2.0275 | - | - | - | 0.013 | 2.028 | 0.013 | - | - | - |
| Tubificida | - | - | - | - | - | - | - | - | - | - | - | - |
