# Supplementary figures and images for: Bridging the biomass data gap: A literature-based Length-Weight Relationship framework for estimating representative dry weights of freshwater invertebrates in Korean rivers
Source: PLoS One. 2026 Jun 23;21(6):e0352157. doi: 10.1371/journal.pone.0352157 (PMC13289862; doi:10.1371/journal.pone.0352157)

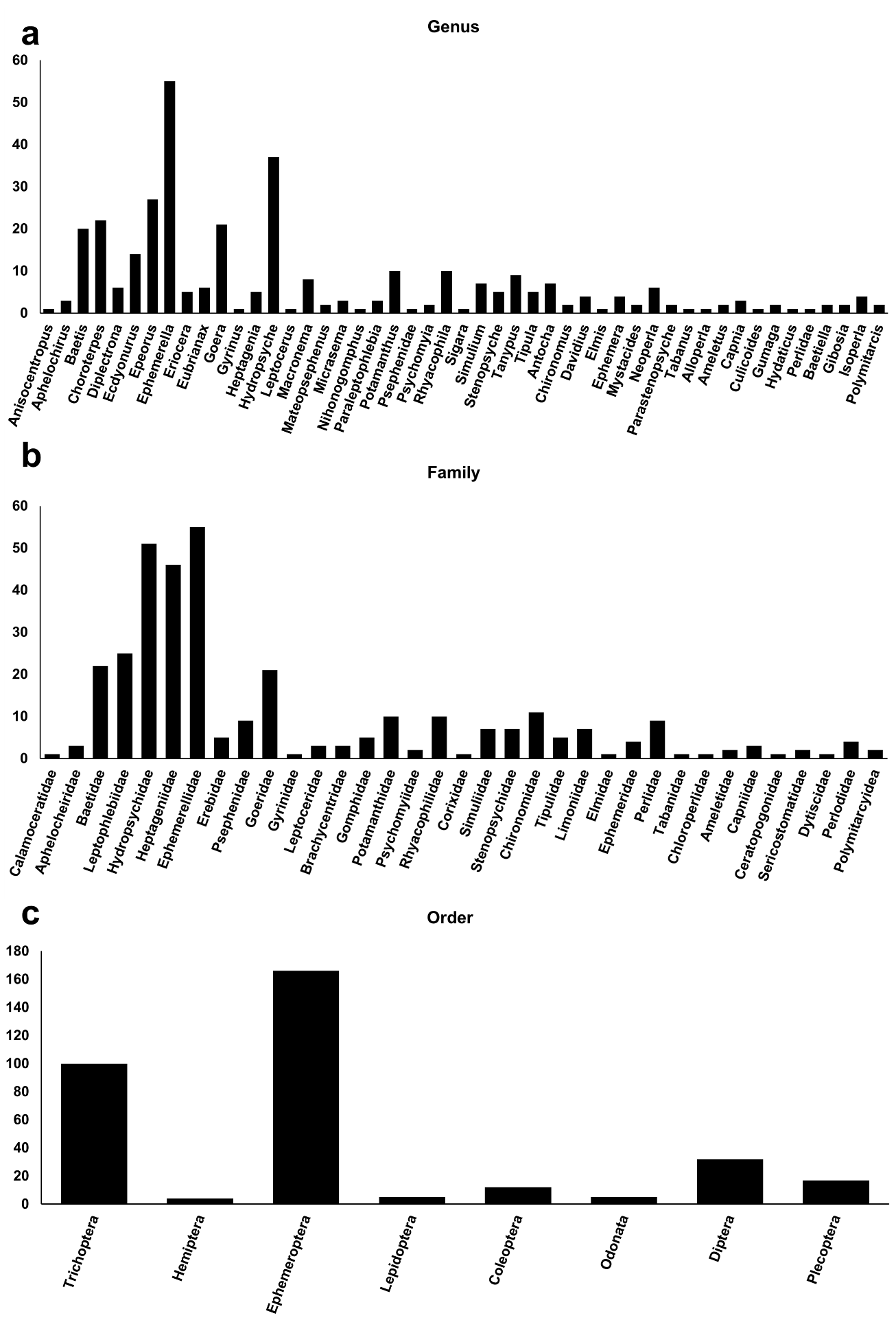

Supplement: S1 Fig — (TIF) [file pone.0352157.s007.tif]

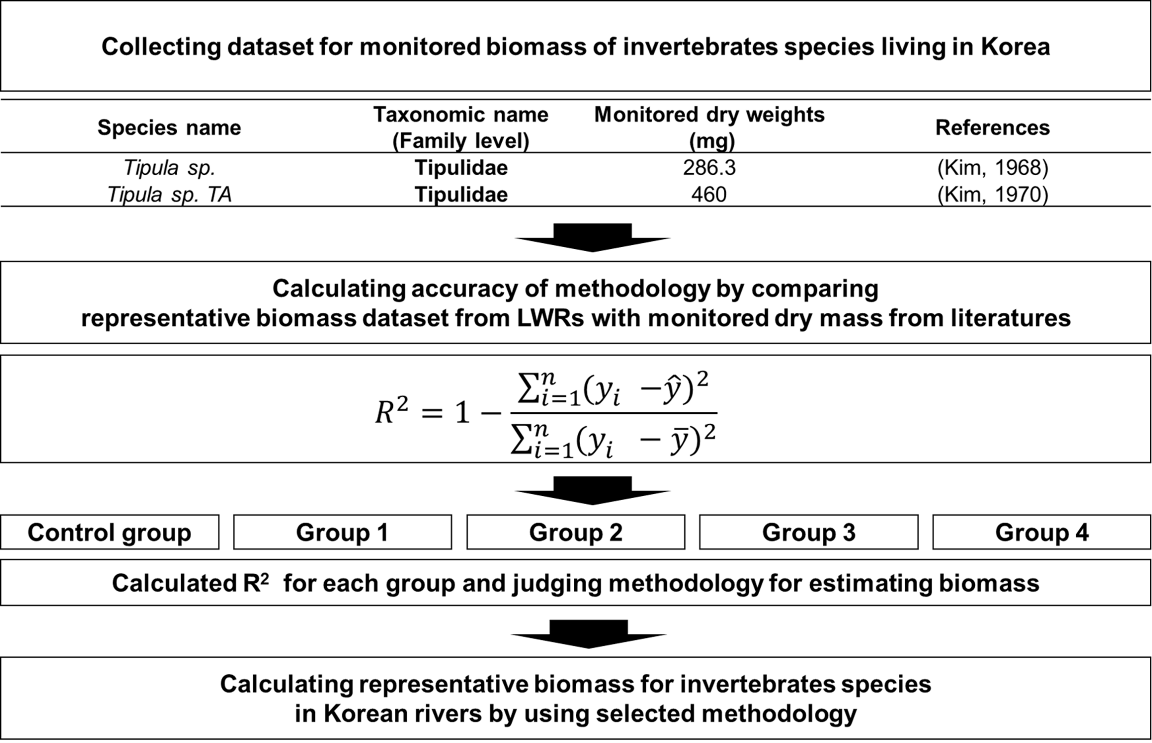

Supplement: S2 Fig — (TIF) [file pone.0352157.s008.tif]
